# Baseline Galectin-3 in Patients with Breast Cancer Scheduled for Anthracycline Therapy: Associations with Cardiac Biomarkers, Left Ventricular Function, and HFA-ICOS Risk Stratification

**DOI:** 10.3390/ijms272110020

**Published:** 2026-11-09

**Authors:** Anna Borowiec, Patrycja Ozdowska, Agnieszka Maria Zebrowska, Magdalena Rosinska, Agata Makowka, Beata Puton, Katarzyna Pogoda, Zbigniew Nowecki, Nicola Maurea, Beata Kotowicz

**Affiliations:** 1 Non-Commercial Clinical Research Outpatient Clinic, Maria Sklodowska-Curie National Research Institute of Oncology, 02-781 Warsaw, Poland; 2 Department of Cancer & Cardio-Oncology Diagnostics, Maria Sklodowska-Curie National Research Institute of Oncology, 02-781 Warsaw, Poland; 3 Unit for Screening Studies in Inherited Cardiovascular Diseases, The Cardinal Stefan Wyszynski National Institute of Cardiology, 04-628 Warsaw, Poland; 4 Digital Medicine Center, Maria Sklodowska-Curie National Research Institute of Oncology, 02-781 Warsaw, Poland; 5 Cancer Biomarker and Cytokines Laboratory Unit, Maria Sklodowska-Curie National Research Institute of Oncology, 02-781 Warsaw, Poland; agata.makowka@nio.gov.pl (A.M.); beata.puton@nio.gov.pl (B.P.); beata.kotowicz@nio.gov.pl (B.K.); 6 Department of Brest Cancer and Reconstructive Surgery, Maria Sklodowska-Curie National Research Institute of Oncology, 02-781 Warsaw, Poland; 7 Division of Cardiology, Istituto Nazionale Tumori-(Istituto di Ricovero e Cura a Carattere Scientifico) IRCCS-Fondazione G. Pascale, 80131 Naples, Italy

**Keywords:** galectin-3, breast cancer, anthracyclines, cardio-oncology, cardiotoxicity, HFA-ICOS, cardiac biomarkers, global longitudinal strain

## Abstract

The clinical role of galectin-3 (Gal-3) as a biomarker in cardio-oncology remains uncertain. This study evaluated baseline Gal-3 concentrations and their associations with established cardiac biomarkers, left ventricular systolic function, and HFA-ICOS cardiovascular risk categories in patients with breast cancer before anthracycline-based chemotherapy. This cross-sectional baseline analysis included 103 patients enrolled in the EMPACT trial. Gal-3 concentrations were evaluated in relation to high-sensitivity cardiac troponin T (hs-cTnT), N-terminal pro-B-type natriuretic peptide (NT-proBNP), left ventricular ejection fraction (LVEF), global longitudinal strain (GLS), HFA-ICOS risk categories, and clinical characteristics. Associations were assessed using Pearson correlation analyses and median regression. Mean baseline Gal-3 concentration was 6.3 ± 2.7 ng/mL. Gal-3 was positively correlated with hs-cTnT (r = 0.384, *p* < 0.001) and NT-proBNP (r = 0.278, *p* = 0.006), but not significantly correlated with LVEF or GLS. Gal-3 concentrations did not differ significantly across HFA-ICOS risk categories (*p* = 0.237). Patients aged ≥65 years had higher Gal-3 concentrations than younger patients (7.1 ± 2.8 vs. 5.7 ± 2.5 ng/mL, *p* = 0.003). In multivariable median regression, age was the only variable significantly associated with Gal-3 among the covariates included in the model (β = 0.82 ng/mL per 10-year increase, 95% CI 0.21–1.43, *p* = 0.009). Associations with hs-cTnT and NT-proBNP were no longer statistically significant after adjustment. In patients with breast cancer before anthracycline therapy, baseline Gal-3 concentrations were associated with age after multivariable adjustment, whereas associations with established cardiac biomarkers were attenuated. No significant associations were observed with LVEF or GLS, and Gal-3 concentrations did not differ significantly across HFA-ICOS risk categories in unadjusted analyses.

## 1. Introduction

Current cardio-oncology guidelines recommend the assessment of cardiac biomarkers, including cardiac troponins (cTn) and natriuretic peptides (B-type natriuretic peptide [BNP] and N-terminal pro-B-type natriuretic peptide [NT-proBNP]), at baseline for cardiovascular risk stratification and throughout cancer treatment for surveillance and the diagnosis of cancer therapy-related cardiac dysfunction (CTRCD) [1]. Although these biomarkers are well established in cardiovascular medicine, their clinical utility in cardio-oncology remains a matter of ongoing debate. In clinical practice, cardiac troponins primarily reflect myocardial injury, whereas natriuretic peptides indicate increased myocardial wall stress; however, neither directly reflects myocardial fibrosis or extracellular matrix remodeling. Consequently, there is growing interest in identifying novel biomarkers capable of detecting subclinical myocardial fibrosis and early adverse cardiac remodeling before structural or functional cardiac impairment becomes clinically apparent.

Galectin-3 is a β-galactoside-binding lectin secreted predominantly by activated macrophages and fibroblasts. It plays a central role in inflammation, fibroblast activation, extracellular matrix remodeling, and myocardial fibrosis [2,3,4,5]. Experimental studies have demonstrated that galectin-3 promotes cardiac remodeling through activation of profibrotic signaling pathways, whereas clinical studies have consistently associated elevated circulating galectin-3 concentrations with adverse outcomes in heart failure, chronic kidney disease, diabetes mellitus, and other cardiovascular disorders [4,6,7,8]. Owing to these biological properties, galectin-3 has emerged as a promising biomarker of myocardial fibrosis and remodeling rather than acute cardiomyocyte injury.

Beyond its established role in cardiovascular disease, galectin-3 is also involved in cancer biology. Galectin-3 is overexpressed in a variety of malignancies, where it contributes to tumor cell proliferation, angiogenesis, invasion, and metastatic spread [7]. Experimental studies have demonstrated that pharmacological inhibition of galectin-3 suppresses tumor growth and metastasis and may enhance the antitumor efficacy of immune checkpoint inhibitors targeting the PD-1/PD-L1 pathway [9]. Furthermore, experimental studies have demonstrated increased galectin-3 (Mac-2) expression in myocardial tissue and macrophages following radiation exposure, suggesting its involvement in radiation-induced cardiac inflammation and fibrosis [10].

In cardio-oncology, however, the clinical significance of galectin-3 remains incompletely understood [11,12]. Experimental studies have demonstrated that anthracycline exposure induces inflammatory, oxidative stress, and profibrotic pathways, whereas galectin-3 has been implicated as a key mediator of myocardial fibrosis and extracellular matrix remodeling, suggesting a potential role in anthracycline-induced cardiac injury [2,4,13,14]. Nevertheless, clinical evidence remains limited and is largely derived from relatively small and heterogeneous patient cohorts. A systematic review and meta-analysis evaluating galectin-3 as a biomarker of cancer therapy-related cardiotoxicity concluded that the available evidence is insufficient to support its clinical utility and that further studies are needed to establish its role in cardio-oncology [11,12]. Furthermore, most published studies have focused on the predictive value of galectin-3 for CTRCD during follow-up, whereas data regarding baseline circulating galectin-3 and its relationship with established cardiac biomarkers before the initiation of potentially cardiotoxic therapy remain scarce. Evaluating galectin-3 before treatment initiation provides an opportunity to characterize its associations with cardiovascular risk indicators independently of anthracycline-induced myocardial injury. This distinction is particularly relevant because circulating galectin-3 may reflect both cardiovascular and noncardiovascular biological processes, including age-related changes, systemic inflammation, and cancer-associated tissue remodeling.

Therefore, the aim of the present analysis was to characterize baseline circulating galectin-3 concentrations in patients with breast cancer enrolled in the EMPACT study before the initiation of anthracycline-based chemotherapy and to evaluate their associations with established cardiac biomarkers (hs-cTnT and NT-proBNP), echocardiographic parameters of left ventricular systolic function (LVEF and GLS), and baseline cardiotoxicity risk assessed using the HFA-ICOS risk assessment tool.

## 2. Results

### 2.1. Baseline Characteristics

**Figure 1:**
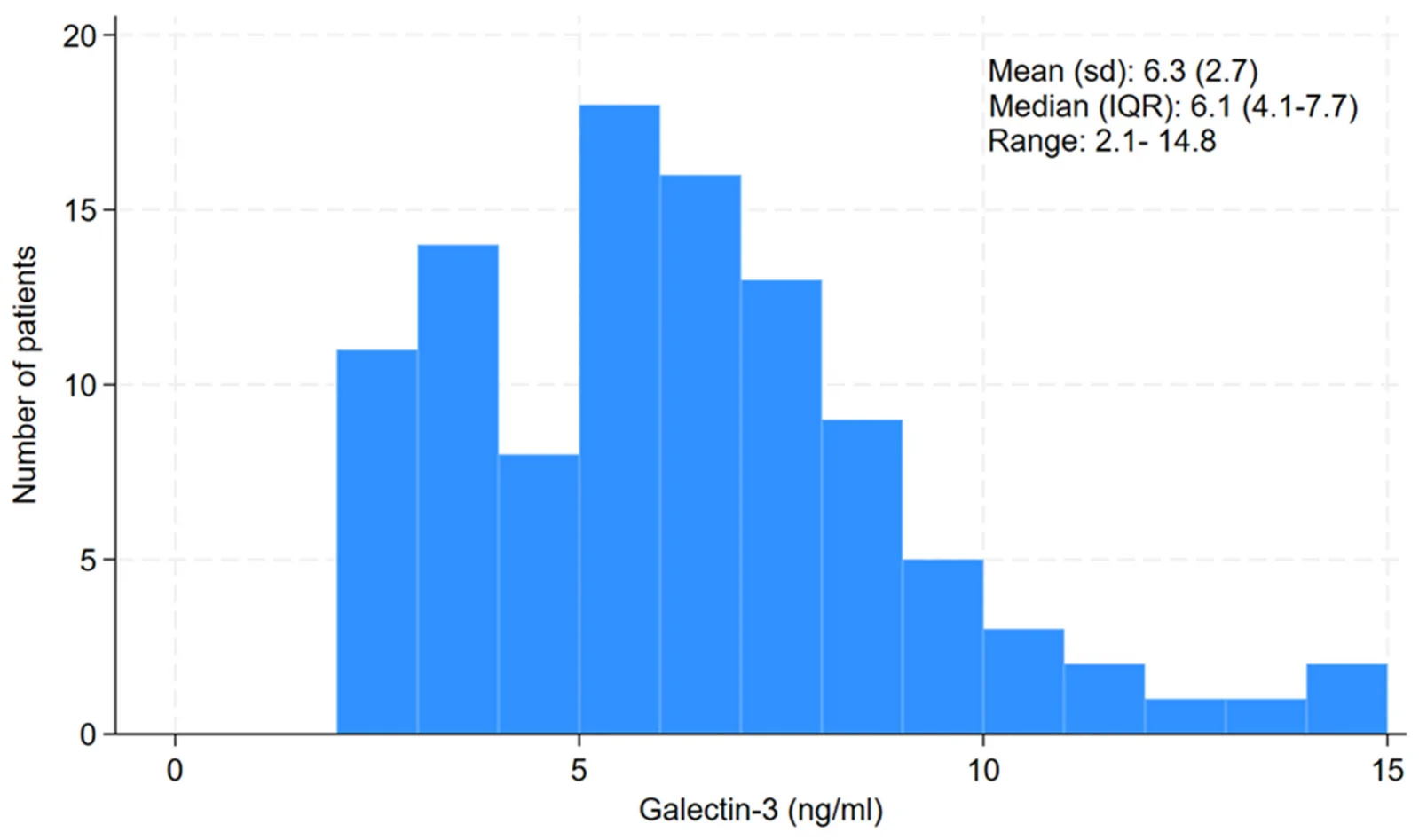
Galectin-3 distribution in the study group.

A total of 103 patients with breast cancer scheduled to receive anthracycline-based chemotherapy were included in the analysis. Triple-negative breast cancer was the most prevalent (54 patients, 52.4%), followed by luminal B (32 patients, 31.1%) and luminal A (17 patients, 16.5%). All patients had non-metastatic breast cancer. None of the participants had a diagnosis of heart failure at baseline. However, 14 patients were classified as NYHA functional class II, indicating mild limitation of physical activity. All participants had a baseline left ventricular ejection fraction (LVEF) ≥50%. The mean baseline circulating galectin-3 concentration was 6.3 ± 2.7 ng/mL, with a median of 6.1 ng/mL (interquartile range [IQR], 4.1–7.7 ng/mL). The distribution of baseline galectin-3 concentrations is shown in Figure 1.

Cardiovascular comorbidities were common, with 70.9% of patients having at least one cardiovascular risk factor. Hypertension and dyslipidemia were present in 45.6% and 47.6% of patients, respectively, while diabetes mellitus was diagnosed in 6.8%. Obesity (BMI ≥ 30 kg/m^2^) was observed in 26.2% of the study population. Baseline cardiac function was preserved, with left ventricular ejection fraction (LVEF) ≥55% in 97.1% of patients. Reduced global longitudinal strain (absolute GLS ≤ 18%) was observed in 34.3% of patients. According to the HFA-ICOS risk stratification tool, 40 patients (38.8%) were classified as low risk, 40 (38.8%) as moderate risk, and 23 (22.3%) as high risk. No patients were classified as very high risk. Elevated baseline high-sensitivity cardiac troponin T (>14 ng/L) and NT-proBNP (>125 pg/mL) were present in 3.1% and 33.3% of patients, respectively, Table 1.

### 2.2. Baseline Galectin-3 Levels According to Clinical Characteristics

Older patients (≥65 years) had significantly higher circulating galectin-3 concentrations than those younger than 65 years (7.1 ± 2.8 vs. 5.7 ± 2.5 ng/mL, *p* = 0.003). Furthermore, patients with elevated baseline cardiac troponin (>14 ng/L) had higher galectin-3 concentrations than those with normal troponin values (10.1 ± 3.9 vs. 6.2 ± 2.7 ng/mL, *p* = 0.041).

No significant differences in galectin-3 concentrations were observed according to NYHA functional class, body mass index, hypertension, dyslipidemia, or baseline NT-proBNP categories (all *p* > 0.05). Similarly, no statistically significant differences were observed across categories of LVEF (*p* = 0.586), absolute GLS (*p* = 0.530), serum creatinine (*p* = 0.570), eGFR (*p* = 1.000), or neutrophil-to-lymphocyte ratio (NLR; *p* = 0.578) categories. In the unadjusted analysis, baseline galectin-3 concentrations did not differ significantly across the low-, moderate-, and high-risk HFA-ICOS categories (*p* = 0.237). Baseline galectin-3 concentrations according to clinical characteristics are presented in Table 2.

### 2.3. Association of Galectin-3 with Cardiac Biomarkers and Echocardiographic Parameters

**Figure 2:**
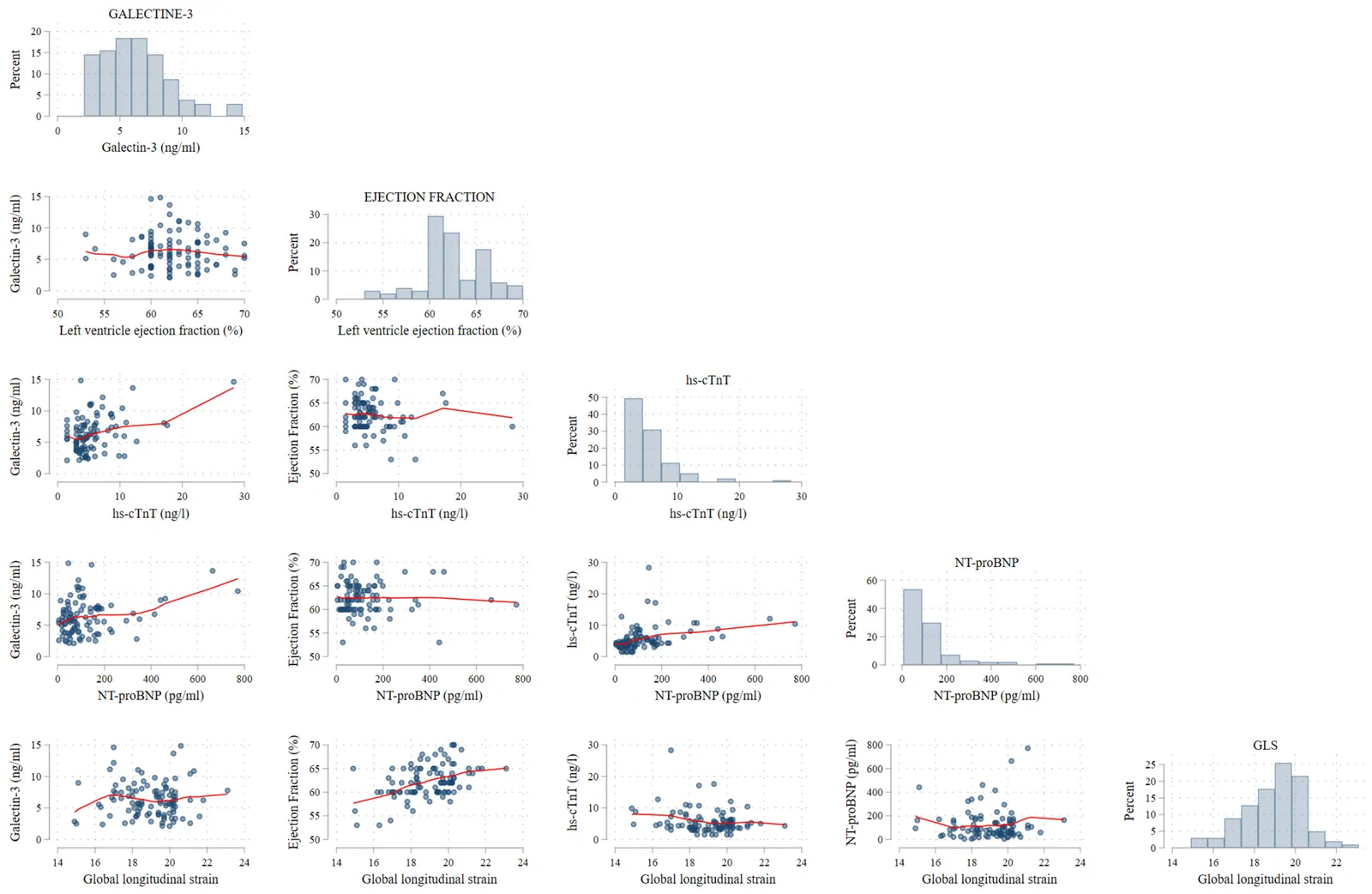
Distributions and pairwise relationships of selected parameters in the study population. Histograms show the distributions of individual variables, while scatter plots illustrate pairwise relationships with locally estimated scatterplot smoothing (LOWESS) curves. LVEF, left ventricular ejection fraction; GLS, global longitudinal strain; hs-cTnT, high-sensitivity cardiac troponin T; NT-proBNP, N-terminal pro-B-type natriuretic peptide.

Pearson correlation analysis demonstrated a moderate positive association between galectin-3 and high-sensitivity cardiac troponin T (hs-cTnT; r = 0.384, 95% CI 0.200–0.542, *p* < 0.001) and a weaker but statistically significant positive association with NT-proBNP (r = 0.278, 95% CI 0.082–0.453, *p* = 0.006). In contrast, no statistically significant correlations were observed between galectin-3 and echocardiographic parameters, including global longitudinal strain (GLS; r = −0.103, 95% CI −0.292 to 0.093, *p* = 0.304) or left ventricular ejection fraction (LVEF; r = −0.048, 95% CI −0.240 to 0.148, *p* = 0.630). A significant positive correlation was also observed between hs-cTnT and NT-proBNP (r = 0.381, *p* < 0.001; Figure 2). Spearman’s rank correlation analysis, performed as a sensitivity analysis, confirmed positive associations between galectin-3 and hs-cTnT (ρ = 0.299, *p* = 0.034) and between galectin-3 and NT-proBNP (ρ = 0.205, *p* = 0.0445), although the correlation coefficients were smaller than those obtained using Pearson’s method. Consistent with the Pearson analysis, no statistically significant correlations were observed between galectin-3 and GLS (ρ = −0.032, *p* = 0.7576) or LVEF (ρ = −0.0296, *p* = 0.7706). The positive association between hs-cTnT and NT-proBNP was also confirmed using Spearman’s rank correlation (ρ = 0.5348, *p* < 0.001). Overall, the Spearman sensitivity analysis yielded findings consistent with the Pearson analysis in terms of the direction and statistical significance of the observed associations, although the correlations between galectin-3 and cardiac biomarkers were attenuated.

### 2.4. Distribution of Galectin-3 According to Baseline Cardiac Biomarker Categories

**Figure 3:**
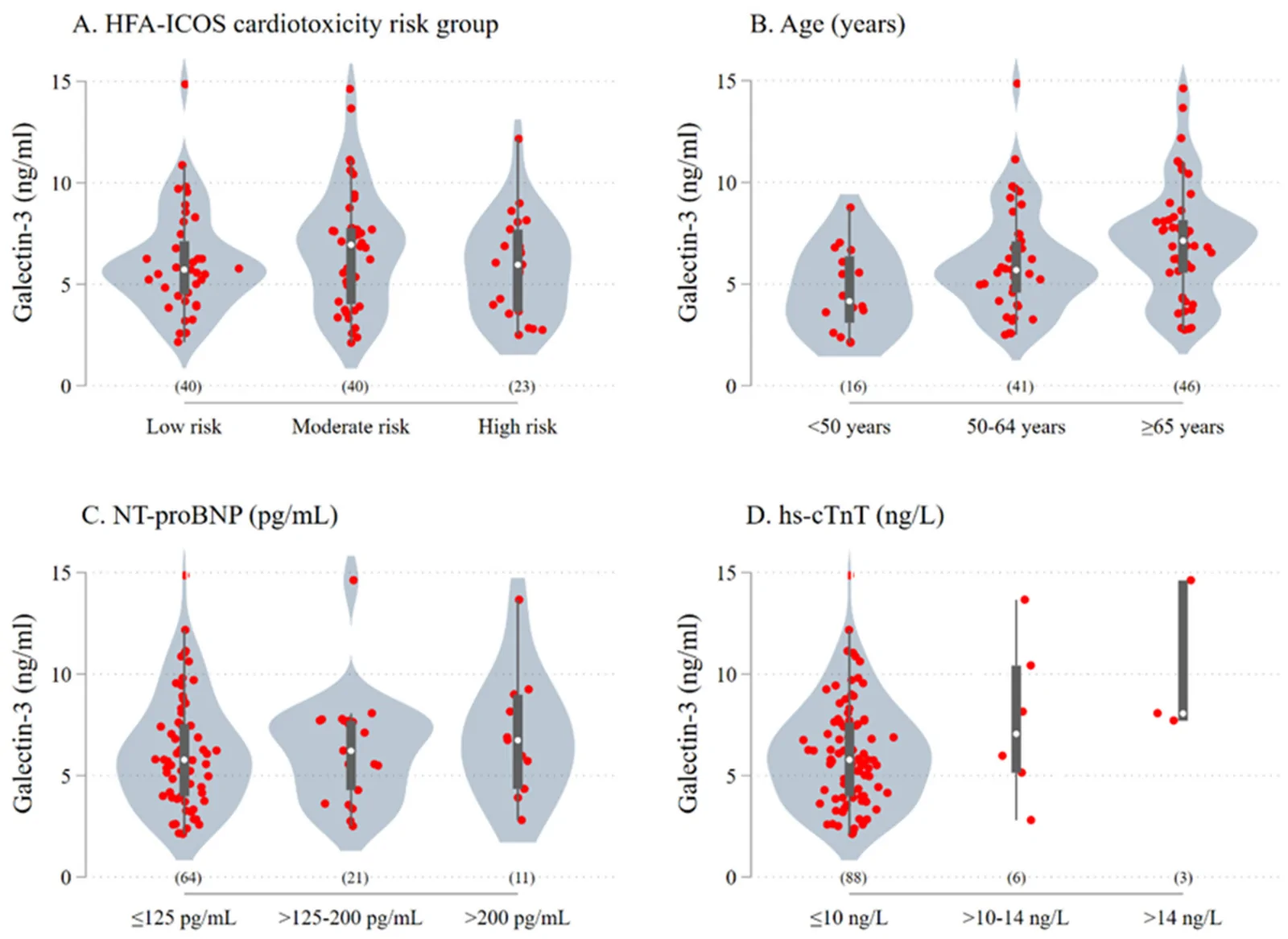
Galectin-3 levels by the HFA ICOS risk group (**A**), age group (**B**), NT-proBNP level (**C**) and hs-cTnT level (**D**). Violin plots show the distribution of the galectin levels in each group; the white dot is the median value, the box corresponds to the interquartile interval and the spikes extend to the upper- and lower-adjacent values. The numbers below each plot indicate the number of patients in each group.

The distribution of galectin-3 according to baseline HFA-ICOS risk category, age group, NT-proBNP concentration, and hs-cTnT concentration is presented in Figure 3. Galectin-3 concentrations increased across age categories, with the highest levels observed in patients aged ≥65 years. Higher galectin-3 concentrations were also observed with increasing hs-cTnT concentrations, particularly among patients with hs-cTnT > 14 ng/L. In contrast, galectin-3 concentrations showed only modest differences across NT-proBNP categories and no consistent gradient across HFA-ICOS risk categories.

### 2.5. Results of Univariable and Multivariable Median Regression

In univariable median regression analyses, older age and higher concentrations of hs-cTnT and NT-proBNP were significantly associated with higher galectin-3 concentrations. Each 10-year increase in age was associated with a 0.75 ng/mL increase in median galectin-3 concentration (β = 0.75, 95% CI 0.28–1.21, *p* = 0.0018). Significant positive associations were also observed for hs-cTnT (β = 3.54 per 10 ng/L increase, 95% CI 1.59–5.49, *p* = 0.0005) and NT-proBNP (β = 0.65 per 100 pg/mL increase, 95% CI 0.10–1.20, *p* = 0.0212). However, after adjustment for age alone, neither hs-cTnT (β = 0.97 per 10 ng/L increase, 95% CI −1.24 to 3.18, *p* = 0.3847) nor NT-proBNP (β = 0.39 per 100 pg/mL increase, 95% CI −0.11 to 0.90, *p* = 0.1253) remained significantly associated with galectin-3 concentrations.

In the multivariable median regression model (*n* = 96), which included serum creatinine as a marker of renal function and the neutrophil-to-lymphocyte ratio (NLR) as an indirect marker of systemic inflammation, age remained the only variable significantly associated with galectin-3 concentrations among the covariates included in the model. Each 10-year increase in age was associated with a 0.82 ng/mL increase in median galectin-3 concentration (β = 0.82, 95% CI 0.21–1.43, *p* = 0.009).

In contrast, neither hs-cTnT (β = 1.42 per 10 ng/L increase, 95% CI −0.66 to 3.49, *p* = 0.1788) nor NT-proBNP (β = 0.38 per 100 pg/mL increase, 95% CI −0.18 to 0.92, *p* = 0.1655) was significantly associated with galectin-3 concentrations after multivariable adjustment. Similarly, no statistically significant associations were observed for serum creatinine (β = −1.32, 95% CI −6.55 to 3.90, *p* = 0.6160) or NLR ≥ 3 versus <3 (β = −0.21, 95% CI −1.30 to 1.11, *p* = 0.7496). GLS, LVEF, obesity, dyslipidemia, hypertension, and type 2 diabetes mellitus were also not significantly associated with galectin-3 concentrations in the multivariable model (Table 3).

In a sensitivity model excluding serum creatinine and NLR, the joint Wald test comparing regression slopes across the 25th, 50th, and 75th conditional percentiles did not reveal statistically significant differences (*p* = 0.913), providing no evidence against the location-shift assumption in that model.

## 3. Discussion

The present study evaluated baseline circulating galectin-3 concentrations in patients with breast cancer before the initiation of anthracycline-based chemotherapy, focusing on their relationships with established cardiac biomarkers, echocardiographic measures of left ventricular systolic function, and HFA-ICOS cardiovascular risk categories. Galectin-3 concentrations were positively associated with age, hs-cTnT, and NT-proBNP in univariable analyses, whereas no statistically significant correlations were observed with LVEF or GLS. Galectin-3 concentrations also did not differ significantly across HFA-ICOS risk categories in the unadjusted comparison. Following multivariable adjustment, age was the only variable significantly associated with galectin-3 among the covariates included in the model.

The main contribution of the present study lies in the characterization of circulating galectin-3 concentrations before anthracycline exposure and their relationships with established cardiovascular biomarkers, echocardiographic parameters, and HFA-ICOS risk categories within the same cohort. Although previous investigations have primarily examined galectin-3 in relation to cancer therapy-related cardiotoxicity, our analysis specifically addresses the interpretation of pretreatment concentrations. Importantly, the observed associations between galectin-3 and hs-cTnT or NT-proBNP were attenuated after adjustment, whereas age remained the only statistically significant variable in the multivariable model. These findings suggest that age-related variation may contribute to the interpretation of baseline galectin-3 concentrations and that unadjusted associations with cardiac biomarkers should be interpreted cautiously. Furthermore, the absence of significant associations with LVEF, GLS, or HFA-ICOS risk categories provides no evidence of additional clinical value for baseline galectin-3 in assessing established cardiovascular risk or subclinical left ventricular dysfunction in this population. Nevertheless, these cross-sectional findings do not exclude a potential prognostic role for galectin-3, which requires evaluation using longitudinal measurements and subsequent cardiovascular outcomes.

The association between galectin-3 and age is consistent with previous observations in population-based cohorts [15]. Circulating galectin-3 concentrations are influenced by demographic and cardiometabolic characteristics, and their relationships with cardiac function may vary across clinical populations. For example, the Gutenberg Health Study demonstrated that associations between galectin-3 and cardiac function differed according to glycemic status, with significant relationships observed primarily among individuals with impaired glucose metabolism [16]. Our findings extend these observations to patients with breast cancer before anthracycline exposure. Although galectin-3 was associated with hs-cTnT and NT-proBNP in univariable analyses, these associations were attenuated and were no longer statistically significant after multivariable adjustment. The relationship with hs-cTnT requires particular caution, as only three participants had concentrations exceeding 14 ng/L and no adjustment for multiple comparisons was performed. In a sensitivity analysis excluding these three participants, the correlation remained statistically significant but was weaker (r = 0.2541, *p* = 0.0135, compared with r = 0.384, *p* < 0.001 in the full sample). Thus, the observations with elevated hs-cTnT contributed to the strength of the association but did not entirely account for it. Nevertheless, given the small subgroup, multiple comparisons, and absence of a statistically significant association after multivariable adjustment, these findings should be considered exploratory and hypothesis-generating.

No statistically significant associations were detected between baseline galectin-3 concentrations and either LVEF or GLS. Although all participants had preserved LVEF and no diagnosis of heart failure at study entry, reduced GLS was observed in a subset of patients, indicating the presence of subclinical abnormalities of left ventricular systolic function. However, the relatively small sample size and restricted range of LVEF values limit the interpretation of these findings. The absence of statistically significant associations therefore does not establish that galectin-3 is unrelated to subclinical myocardial dysfunction. Previous studies have also reported inconsistent relationships between galectin-3 and echocardiographic parameters. In patients with cardiac light-chain amyloidosis, galectin-3 was not significantly associated with cardiac troponin I, NT-proBNP, or LVEF [17]. Similarly, in systemic sclerosis, galectin-3 showed no significant correlation with NT-proBNP or LVEF, although an association with GLS was observed [18]. These findings suggest that the relationship between circulating galectin-3 and myocardial function may depend on the underlying clinical condition.

An important consideration is that circulating galectin-3 is not a cardiac-specific biomarker. It is expressed in various tissues and cell types and participates in biological processes involving inflammation, tissue remodeling, fibrosis, and cancer progression. In patients with active malignancy, circulating galectin-3 concentrations may therefore reflect tumor-related processes, systemic inflammation, and other noncardiac influences, in addition to potential cardiovascular contributions. Consequently, circulating galectin-3 should not be interpreted as a direct or specific measure of myocardial fibrosis. This biological complexity may partly explain the absence of consistent associations with cardiac biomarkers and echocardiographic parameters. However, the relative contributions of cardiac and noncardiac sources could not be determined in the present study.

The clinical role of galectin-3 in cardio-oncology remains uncertain. In a study of breast cancer survivors, galectin-3 concentrations did not differ significantly between women previously treated with anthracyclines and those without anthracycline exposure [19]. Furthermore, a systematic review and meta-analysis of eight studies involving 1979 patients found no statistically significant overall change in galectin-3 concentrations following cancer therapy and no statistically significant pooled association with cancer therapy-related cardiotoxicity [12]. However, the available evidence remains limited and heterogeneous, particularly regarding galectin-3 concentrations before the initiation of potentially cardiotoxic treatment. Our study contributes to this evidence by examining pretreatment galectin-3 concentrations alongside established cardiac biomarkers, LVEF, GLS, and HFA-ICOS risk categories within the same cohort. In particular, the comparison across HFA-ICOS categories provides information on the relationship between galectin-3 and an established multidimensional assessment of baseline cardiovascular toxicity risk. The absence of significant differences in galectin-3 concentrations across HFA-ICOS risk categories warrants careful interpretation. The HFA-ICOS classification integrates multiple clinical and cardiovascular risk factors, whereas galectin-3 is involved in biological processes related to inflammation and fibrosis. Consequently, galectin-3 concentrations may not directly reflect the overall cardiovascular risk profile represented by HFA-ICOS categories. Moreover, the relatively small number of patients, particularly in the high-risk group, may have limited the ability to detect differences.

In conclusion, this baseline analysis provides additional evidence on the relationships between circulating galectin-3, established cardiac biomarkers, echocardiographic parameters, and HFA-ICOS risk categories in patients with breast cancer before anthracycline treatment. Age was the only variable significantly associated with galectin-3 among the covariates included in the multivariable model, while no significant correlations were detected with LVEF or GLS and no significant differences were observed across HFA-ICOS risk categories. These findings do not exclude a potential role for galectin-3 in cardio-oncology but underscore the need for larger prospective studies incorporating serial biomarker measurements, comprehensive assessment of potential confounders, and longitudinal cardiovascular outcomes.

### Study Limitations

Several limitations should be acknowledged. First, this exploratory, non-prespecified secondary analysis of baseline data from the EMPACT trial included a relatively small cohort of 103 patients with nonmetastatic breast cancer, potentially limiting statistical power and the stability of multivariable estimates. All participants had preserved baseline LVEF, restricting the range of ventricular systolic function and potentially limiting the detection of associations with galectin-3, including those related to subclinical myocardial dysfunction. These findings may therefore not be generalizable to patients with other malignancies, metastatic disease, established cardiovascular disease, reduced LVEF, or more pronounced renal impairment. Second, only three patients had hs-cTnT concentrations >14 ng/L, limiting the interpretation of findings in this subgroup. Although the correlation between galectin-3 and hs-cTnT remained statistically significant after excluding these patients, its magnitude was reduced. Third, eGFR values were available for only 34 patients (33.0%), whereas serum creatinine concentrations were available for 99 patients (96.1%). Consequently, serum creatinine was used as a marker of renal function in the multivariable analysis. However, serum creatinine and the neutrophil-to-lymphocyte ratio, included as an indirect marker of systemic inflammation, may not fully account for renal function and inflammatory status, respectively, and residual confounding cannot be excluded. Finally, galectin-3 was assessed at a single pretreatment time point. Its circulating concentrations may reflect both cardiovascular and cancer-related biological processes, which could not be distinguished in this study. Furthermore, the analysis did not assess longitudinal changes in galectin-3 or its association with subsequent cancer therapy-related cardiac dysfunction. Therefore, the present findings cannot establish the prognostic utility of galectin-3 in cardio-oncology.

## 4. Materials and Methods

### 4.1. Study Structure

The present analysis was conducted within the EMPACT (EMPAgliflozin in the Prevention of Chemotherapy-related CardioToxicity) study, a prospective, multicenter, randomized, double-blind, placebo-controlled phase III clinical trial designed to evaluate the efficacy and safety of empagliflozin in the prevention of cancer therapy-related cardiac dysfunction (CTRCD) in patients receiving anthracycline-based chemotherapy. The present study represents an analysis of baseline data from patients with breast cancer enrolled in EMPACT. Only measurements obtained before the initiation of anthracycline chemotherapy and before administration of the randomized study treatment were included in the current analysis. Galectin-3 measurements at predefined study visits were included in the original EMPACT trial protocol. However, the specific analyses presented in this manuscript were not prespecified and were conducted as an exploratory secondary analysis of baseline data collected before anthracycline exposure. The analysis focused on baseline circulating galectin-3 concentrations and their relationships with clinical characteristics, cardiovascular risk factors, HFA-ICOS cardiotoxicity risk categories, hs-cTnT, NT-proBNP, LVEF, and GLS.

The EMPACT study is sponsored by the Maria Skłodowska-Curie National Research Institute of Oncology in Warsaw, Poland, and is funded by the Polish Medical Research Agency (no. 2021/ABM/03/00012). The trial is registered at ClinicalTrials.gov (NCT05271162), EudraCT (2022-003043-10), and the Clinical Trials Information System (EU CT No. 2024-515495-13-00). The study is conducted in accordance with the principles of the Declaration of Helsinki and Good Clinical Practice guidelines. The conduct of the study was approved by an independent Ethics Committee at the Maria Sklodowska-Curie National Research Institute of Oncology in Warsaw (approval no. 75/2022), and all participants gave their informed written consent before entering the study.

### 4.2. Study Population

The analysis included 103 consecutive patients with breast cancer who were scheduled to receive anthracycline-based chemotherapy. The main eligibility criteria for the EMPACT trial included age ≥18 years, Eastern Cooperative Oncology Group (ECOG) performance status of 0–2, a diagnosis of cancer with an indication for systemic anticancer treatment including a planned cumulative anthracycline dose of ≥240 mg/m^2^ of doxorubicin or ≥360 mg/m^2^ of epirubicin, no history of heart failure, and preserved left ventricular systolic function, defined as a baseline left ventricular ejection fraction (LVEF) ≥50%. The principal exclusion criteria included a history of heart failure, baseline LVEF <50%, significant valvular heart disease, previous chemotherapy or thoracic radiotherapy, symptomatic hypotension or systolic blood pressure <100 mmHg, severe hepatic dysfunction, severe renal impairment defined as an estimated glomerular filtration rate <20 mL/min/1.73 m^2^ or the need for dialysis, a history of ketoacidosis, conditions potentially interfering with study drug absorption, an anticipated life expectancy of <1 year, treatment with a sodium–glucose cotransporter 2 (SGLT2) inhibitor within 3 months before enrollment, pregnancy or breastfeeding, and contraindications to cardiovascular magnetic resonance imaging.

At baseline, demographic characteristics, cardiovascular history and risk factors, concomitant diseases, and clinical status were recorded. All participants underwent a comprehensive cardiovascular assessment, including transthoracic echocardiography with measurement of LVEF and global longitudinal strain (GLS), electrocardiography, and laboratory assessment including high-sensitivity cardiac troponin T (hs-cTnT), N-terminal pro-B-type natriuretic peptide (NT-proBNP), and galectin-3. Baseline cardiotoxicity risk was assessed according to the Heart Failure Association–International Cardio-Oncology Society (HFA-ICOS) risk stratification tool.

For the purposes of the present analysis, only pretreatment measurements were considered; therefore, the results were not influenced by anthracycline exposure or by randomization to empagliflozin or placebo. The objective of this analysis was to characterize circulating galectin-3 concentrations before anthracycline treatment and to assess their associations with clinical characteristics, established cardiac biomarkers, echocardiographic measures of left ventricular function, and baseline HFA-ICOS cardiotoxicity risk.

### 4.3. Echocardiographic Assessment

Transthoracic echocardiography was performed at baseline, before the initiation of anthracycline-based chemotherapy and before administration of the randomized study treatment. Echocardiographic examinations included standard assessment of cardiac structure and function, with particular emphasis on left ventricular systolic function. All examinations were performed using a Vivid E95 ultrasound system (GE Healthcare, Horten, Norway). Left ventricular ejection fraction (LVEF) was assessed using the biplane Simpson method from apical four- and two-chamber views. Left ventricular global longitudinal strain (GLS) was assessed using two-dimensional speckle-tracking echocardiography from standard apical views, with Automated Function Imaging software (AFI, version 3.0, GE Healthcare). GLS was analyzed using absolute values, with lower absolute values indicating worse myocardial deformation. All echocardiographic examinations and measurements were performed in accordance with current recommendations of the European Association of Cardiovascular Imaging (EACVI). Echocardiographic examinations and GLS measurements were performed and analyzed independently at each participating center. The reproducibility of echocardiographic measurements was assessed. For the present analysis, baseline LVEF and GLS measurements were used to investigate their relationships with circulating galectin-3 concentrations. All participants had preserved left ventricular systolic function at study entry, as required by the EMPACT eligibility criteria (LVEF ≥ 50%).

### 4.4. Biomarker Assessment

Plasma concentrations of high-sensitivity cardiac troponin T (hs-cTnT) and N-terminal pro-B-type natriuretic peptide (NT-proBNP) were measured at baseline in a central laboratory using standardized laboratory methods. Blood samples were collected before the initiation of anthracycline-based chemotherapy. For the present analysis, hs-cTnT and NT-proBNP were analyzed both as continuous variables and according to clinically relevant cut-off values. An hs-cTnT concentration >14 ng/L and an NT-proBNP concentration >125 pg/mL were considered elevated. Plasma hs-cTnT and NT-proBNP concentrations were measured using electrochemiluminescence immunoassays (Elecsys, Roche Diagnostics, Mannheim, Germany).

#### Galectin-3 Measurement

Serum galectin-3 concentrations were quantified using a commercial sandwich enzyme-linked immunosorbent assay (ELISA; Human Galectin-3 Quantikine ELISA Kit, R&D Systems, Minneapolis, MN, USA; catalog no. DGAL30), according to the manufacturer’s instructions. In this assay, galectin-3 present in the sample is captured by a monoclonal antibody immobilized on the microplate and subsequently detected using an enzyme-linked conjugate. Venous blood samples were centrifuged at 1000× *g* for 15 min, and the separated serum was aliquoted and stored at ≤−80 °C for 8–12 months until analysis; each aliquot was thawed only once, immediately before the assay. All serum samples were diluted two-fold according to the manufacturer’s protocol, and the measured concentrations were multiplied by the dilution factor to obtain the final galectin-3 concentrations. A serum volume of 50 μL was used for each determination.

Samples were analyzed in several plates on different days using kits of the same lot. Each plate contained 40 patient samples together with a calibration curve, all assayed in duplicate; duplicate measurements were accepted if the coefficient of variation (CV) was ≤10%, and the mean of the duplicates was used for analysis. Following substrate addition, absorbance was measured at 450 nm using a microplate reader. Data were analyzed with Gen5 software (version Gen5 3.17.17) (BioTek Instruments, Agilent Technologies, Winooski, VT, USA): on each plate, the mean of duplicate readings was calculated after subtraction of the mean zero-standard optical density, and galectin-3 concentrations were interpolated from a four-parameter logistic (4PL) standard curve fitted to the calibrators. The assay range was 0.313–10 ng/mL, with a manufacturer-reported analytical sensitivity of 0.085 ng/mL. Intra-assay and inter-assay CVs for serum, as reported by the manufacturer, were 3.5–3.8% and 5.8–6.3%, respectively. All reported galectin-3 concentrations were within the assay calibration range after accounting for the two-fold sample dilution. The highest observed concentration (14.8 ng/mL) corresponded to 7.4 ng/mL in the diluted sample; therefore, no additional dilution or reassessment was required due to exceeding the upper calibration limit.

### 4.5. HFA-ICOS Cardiotoxicity Risk Assessment

Baseline risk of cancer therapy-related cardiovascular toxicity was assessed before the initiation of anthracycline-based chemotherapy using the Heart Failure Association–International Cardio-Oncology Society (HFA-ICOS) risk assessment tool, as recommended by international cardio-oncology guidelines (1). Risk stratification was based on cardiovascular history, cardiovascular risk factors, previous exposure to potentially cardiotoxic cancer therapies, lifestyle-related risk factors, and baseline cardiac assessment, including cardiac biomarkers and echocardiographic parameters, as applicable. According to the HFA-ICOS risk assessment tool for patients receiving anthracyclines, patients were classified into one of four baseline cardiotoxicity risk categories: low, moderate, high, or very high risk. For the present analysis, baseline galectin-3 concentrations were compared across HFA-ICOS risk categories to investigate whether circulating galectin-3 was associated with the established baseline cardiotoxicity risk profile. Galectin-3 was not included as a component of the HFA-ICOS risk score and was therefore analyzed as an independent exploratory biomarker.

### 4.6. Data Acquisition and Statistical Analysis

#### 4.6.1. Data Acquisition and Quality Control

All measurements were performed according to routine diagnostic standards. Critical measurements (echocardiography, biomarkers) were reviewed and coded by a trained member of the study team to ensure the comparability of the results. Clinical data was captured by manual entry into electronic case report forms (eCRF) and laboratory data extracted from the Hospital Information System (HIS). The data was validated by the clinical team to eliminate inconsistent, missing and outlying values. In particular, the extreme values were reviewed for clinical plausibility and consistency of measurement units. Missing numeric values were reviewed for typographical or formatting errors in the original text entries that could have caused failures during text-to-numeric conversion. Confirmed errors were corrected against source records, clinically plausible extreme values were retained, and values that remained unavailable were treated as missing.

#### 4.6.2. Statistical Analysis

Galectin-3 concentrations were summarized using means and standard deviations (SDs), as well as medians and interquartile ranges (IQRs), and analyzed in relation to patient characteristics, established risk factors for cardiotoxicity, and cardiac biomarkers. Given the skewed distribution of galectin-3 concentrations, unadjusted between-group comparisons were performed using the nonparametric k-sample median test, with exact inference applied when group sizes were small. Pearson correlation coefficients were calculated to assess linear associations between galectin-3 concentrations and cardiac biomarkers or echocardiographic parameters. The statistical significance of Pearson correlations was assessed using Fisher’s method. Spearman’s rank correlation coefficients were additionally calculated as a sensitivity analysis to evaluate the robustness of the observed associations to skewed distributions and potentially influential observations.

No adjustment for multiple comparisons was applied. Given the exploratory nature of the analyses and the assessment of multiple factors in univariable comparisons, nominally significant findings, particularly those with *p*-values close to 0.05, were interpreted cautiously and considered hypothesis-generating rather than confirmatory. Multivariable associations were assessed using median regression (quantile regression at τ = 0.50), with galectin-3 concentration as the dependent variable [20]. Covariates were selected based on previously reported associations with galectin-3 concentrations. Given the relatively small sample size, additional models adjusted for age alone were also examined. The location-shift assumption underlying inference based on independent and identically distributed (i.i.d.) errors was assessed using simultaneous quantile regression at the 25th, 50th, and 75th conditional percentiles (Stata command ‘sqreg’). A joint Wald test was performed to assess the equality of regression slopes across these quantiles, allowing intercepts to differ, using a variance–covariance matrix estimated from 5000 bootstrap replications. Regression coefficients were interpreted as adjusted differences in the conditional median of galectin-3 concentrations, holding the other covariates constant. Standard errors for the median regression model were estimated under the assumption of i.i.d. errors. The composite HFA–ICOS risk score was not included in the multivariable models because individual components of the score were already included as covariates, thereby avoiding redundancy and limiting potential multicollinearity. Missing data were not imputed. Multivariable regression was performed using complete-case analysis. Although the availability of individual variables differed, missing observations overlapped across patients, resulting in 96 of 103 participants (93.2%) with complete data for all covariates included in the final model. Univariable analyses included all available observations for the variables involved in each comparison. All statistical analyses were performed using Stata/SE version 17.0 (StataCorp LLC, College Station, TX, USA).

## 5. Conclusions

In this exploratory baseline analysis of patients with breast cancer scheduled to receive anthracycline-based chemotherapy, age was the only covariate significantly associated with galectin-3 concentrations in the reported multivariable model. Unadjusted associations with hs-cTnT and NT-proBNP were attenuated after adjustment, and no statistically significant associations with LVEF, GLS, or HFA-ICOS risk categories were detected.

## Figures and Tables

**Table 1:** Baseline characteristics of the study group (N = 103).

Demographic Characteristics	*n*/Total Non-Missing (%)
Age < 65 years	57/103 (55.3)
Age ≥ 65 years	46/103 (44.7)
Age (years), mean (sd), median [IQR], range	63.3 (13.3), 62 (54–76), 33–91
**Cardiovascular history and risk factors**	
Hypertension	47/103 (45.6)
Dyslipidemia	49/103 (47.6)
Peripheral vascular disease	3/103 (2.9)
Diabetes mellitus type 2	7/103 (6.8)
BMI ≥ 30 kg/m^2^	27/103 (26.2)
BMI (kg/m2), mean (sd), median [IQR], range	27.5 (4.8), 26.8 [24.4–30.1], 19.7–45.8
**Baseline renal function**	
Creatinine ≥ 1 mg/dL	3/99 (3.0)
Creatinine (mg/dL), mean (sd), median [IQR], range	0.7 (0.13), 0.7 [0.65–0.8], 0.5–1.25
eGFR < 90 (mL/min/1.73 m^2^)	14/34 (41.2)
eGFR (mL/min/1.73 m^2^), mean (sd), median [IQR], range	90.3 (12.7), 91 (80–99), 68–123
**Inflammatory status at baseline**	
Neutrophil-to-lymphocyte ratio (NLR) ≥3.0	35/99 (35.4)
Neutrophil-to-lymphocyte ratio (NLR)	3.6 (6.2), 2.6 [2.1–3.5], 0.91–62.4
**Baseline echocardiography**	
LVEF ≥ 55%	99/102 (97.1)
LVEF 50–54%	3/102 (2.9)
LVEF (%), mean (sd), median [IQR], range	62.3 (3.4), 62 [60–65], 53–70
GLS > 18%	67/102 (65.7)
GLS ≤ 18%	35/102 (34.3)
GLS (absolute value), mean (sd), median [IQR], range	19.0 (1.5), 19.3 [18–20.1], 14.9–23.1
**Baseline biomarkers**	
hs-cTnT > 14 ng/L	3/97 (3.1)
hs-cTnT (ng/L), mean (sd), median [IQR], range	5.6 (3.8), 4.5 [3.6–6.2], 1.5–28.3
NT-proBNP > 125 pg/mL	32/96 (33.3)
NT-proBNP (pg/mL), mean (sd), median [IQR], range	122.1 (128.2), 83.6 [48.5–157.0], 5–772
**HFA-ICOS risk group**	
Low risk	40/103 (38.8)
Moderate risk	40/103 (38.8)
High risk	23/103 (22.3)

**Table 2:** Baseline galectin-3 concentrations according to clinical characteristics.

Factor	Level	*n*	Galectin-3 Concentration (ng/mL)		*p*-Value
			Mean (SD)	Median (IQR)	
Age group	<65	57	5.7 (2.5)	5.5 (3.9–6.8)	0.003
	65+	46	7.1 (2.8)	7.1 (5.6–8.1)	
HFA-ICOS risk group	Low risk	40	6.1 (2.5)	5.7 (4.5–7.1)	0.237
	Moderate risk	40	6.8 (3.0)	6.9 (4.0–7.8)	
	High risk	23	5.8 (2.4)	6.0 (3.7–7.7)	
NYHA classification	1	89	6.1 (2.5)	5.8 (4.2–7.6)	0.214
	2	14	7.4 (3.6)	7.5 (3.6–10.6)	
GLS	>18%	67	6.1 (2.4)	5.7 (4.0–7.7)	0.53
	≤18%	35	6.6 (3.2)	6.5 (4.3–8.1)	
LVEF	55%+	99	6.3 (2.7)	6.0 (4.0–7.7)	0.586
	50–54%	3	6.9 (1.9)	6.7 (5.1–9.0)	
NT-proBNP (pg/L)	≤125	64	6.2 (2.7)	5.8 (4.0–7.5)	0.504
	>125	32	6.5 (2.8)	6.5 (4.3–7.7)	
hs-cTnT (ng/L)	≤14	94	6.2 (2.7)	5.8 (4.0–7.7)	0.041
	>14	3	10.1 (3.9)	8.1 (7.7–14.6)	
BMI (kg/m^2^)	<30	76	6.5 (2.7)	6.0 (4.4–7.7)	0.356
	≥30	27	5.9 (2.8)	6.1 (2.8–7.4)	
Dyslipidemia	No	54	6.7 (2.7)	6.5 (4.8–8.3)	0.077
	Yes	49	5.8 (2.6)	5.8 (3.9–7.4)	
Hypertension	No	56	6.2 (2.8)	5.8 (4.3–7.4)	0.4
	Yes	47	6.4 (2.6)	6.7 (3.9–8.5)	
Creatinine (mg/dL)	<1	96	6.3 (2.7)	6.1 (4.1–7.7)	0.57
	≥1	3	6.6 (5.0)	5.1 (2.4–12.2)	
eGRF (mL/min/1.73 m^2^)	<90	14	7.2 (3.9)	5.6 (4.3–9.7)	1
	≥90	20	5.6 (2.4)	5.6 (3.5–7.8)	
Neutrophil-to-lymphocyte ratio (NLR)	<3	64	6.4 (2.7)	6.2 (4.2–7.9)	0.578
	≥3	35	6.2 (2.8)	5.8 (3.7–7.7)	

**Table 3:** Results of univariable, age-adjusted and multivariable median regression.

	*n* *	Coefficient (95% Confidence Interval), Univariate Models	*p*-Value	Coefficient (95% Confidence Interval), Age-Adjusted Models **	*p*-Value	Coefficient (95% Confidence Interval), Multivariate Model, *n* = 96	*p*-Value
Age (per 10-year increase)	103	0.75 (0.28–1.21)	0.0018	-		0.82 (0.21–1.43)	0.009
hs-cTnT (per 10 ng/L increase)	97	3.54 (1.59–5.49)	0.0005	0.97 (−1.24–3.18)	0.3847	1.42 (−0.66–3.49)	0.1788
NT-proBNP (per 100 pg/mL increase)	96	0.65 (0.1–1.2)	0.0212	0.39 (−0.11–0.9)	0.1253	0.38 (−0.18–0.92)	0.1655
GLS (per 1% increase)	102	0.08 (−0.42–0.57)	0.7559	0.08 (−0.35–0.5)	0.7206	−0.02 (−0.48–0.52)	0.9329
LVEF (per 5% increase)	102	−0.35 (−1.4–0.7)	0.5152	0.05 (−0.9–1)	0.9217	−0.13 (−1.15–0.88)	0.7936
Creatinine (mg/dl)	99	0.22 (−5.09–5.53)	0.9333	−1.25 (−6.01–3.51)	0.6029	−1.32 (−6.55–3.90)	0.6160
NLR (≥3 vs. <3)	99	−0.46 (−1.94–1.02)	0.5375	0.17 (−1.02–1.35)	0.7785	−0.21 (−1.3–1.11)	0.7496
BMI (≥30 vs. <30)	103	−0.03 (−1.54–1.49)	0.9729	−0.46 (−2.01–1.08)	0.5525	−0.46 (−1.96–1.04)	0.5413
Dislipidemia (Yes vs. No)	103	−0.89 (−2.3–0.51)	0.2096	−0.87 (−2.03–0.29)	0.1418	−1.13 (−2.44–0.18)	0.0906
Hypertension (Yes vs. No)	103	0.89 (−0.43–2.22)	0.1837	−0.35 (−1.52–0.81)	0.5482	−0.65 (−2.04–0.73)	0.3499
Type II diabetes (Yes vs. No)	103	−0.48 (−2.03–1.07)	0.5424	0.01 (−1.42–1.44)	0.989	−0.10 (−1.49–1.29)	0.8906

* *n*—number of observations used in each of univariate and age-adjusted models; ** age-adjusted models include the analysed factor and age linear effect.

## Data Availability

The datasets used and/or analysed during the current study are available from the corresponding author on reasonable request.

## Abbreviations

CTRCD	cancer therapy-related cardiac dysfunction
CTR-CVT	cancer therapy-related cardiovascular toxicity
HFA-ICOS	Heart Failure Association–International Cardio-Oncology Society
GLS	left ventricular peak systolic global longitudinal strain
hs-cTnT	high-sensitivity cardiac troponin T
NT-proBNP	N-terminal pro-B-type natriuretic peptide
eCRF	electronic case report forms
HIS	Hospital Information System
LVEF	left ventricle ejection fraction
CVD	cardiovascular disease
Gal-3	Galectin-3
